# Surgical Treatment of Shoe-boil

**Published:** 1899-04

**Authors:** 


Surgical Treatment of Shoe-boil. The word "shoe-boil" may be said to include a number of pathological conditions, such as serous cysts, hæmatoma, abscess, and fibrous tumors. As to the last variety of tumor, the periphery may be well defined, or it may be œdematous and attached to the surrounding tissues.

The treatment of these various conditions is an everyday occurrence to every veterinarian.

Serous cysts may disappear by absorption from the use of stimulating applications. This process is, however, slow and tedious, often unsuccessful, and if there is no evidence of a prompt improvement, the best treatment is the opening of the sac with the bistoury and allowing the cavity to cicatrize. There is no danger of infection from incising a cyst if ordinary antisepsis be employed. This practice I have followed in the treatment of all serous cysts, whether situated on the inside of the knee, elbow, withers, stifle, or buttock. It saves time, and the walls of the cavity will unite without much suppuration.

Abscesses and blood tumors require their usual treatment.

It is more especially of the hard fibrous tumors of which I wish to speak. The treatment that is the safest and of shortest duration is often the last one chosen. The elastic ligature setons, caustics, and the actual cautery are often experimented with. The first may be used when the tumor is very decidedly pediculated, which does not exist very often ; if not pediculated it requires a long time and a large, ulcerating surface remains. The actions of caustics cannot always be controlled and, like the elastic ligature, they leave a large ulcerating surface, which, at the point of the elbow, cicatrizes only with great difficulty. The treatment which, in all the ordinary cases observed, I have found most efficacious is simple excision, as in all other kinds of tumors, even when these are voluminous. The anatomy of the point of the elbow is such that the operation can be performed with safety, and whatever hemorrhage may take place can be arrested by the ordinary means. Sometimes, with the use of cocaine, it may not even be necessary to cast the animal. If this treatment be resorted to at the beginning time will be saved and the result will be more satisfactory. The lips of the incision can be placed quite well into apposition. This will leave a small cavity to cicatrize without the formation of a large granulating sore difficult to heal. Primary union is not expected, but at some points at least the lips will unite before the sutures are torn and the wound is prevented from gaping. Drainage is secured by means of a perforated rubber tube projecting from both commissures of the incision. Large tumors can in this way be successfully treated in a comparatively short time without local blemish, and more effectively than with the other methods often recommended.

